# Heteroatomic Sites on Carbon: Thermal CO_2_ Activation and Transformation by Metal‐Free Catalysis

**DOI:** 10.1002/cssc.202501052

**Published:** 2025-08-20

**Authors:** Peng Zhang, Chaoan Liang, Tao Du, Jiali Sun, Bohao Pang, Saskia Heumann, Yuxiao Ding

**Affiliations:** ^1^ State Key Laboratory of Low Carbon Catalysis and Carbon Dioxide Utilization Lanzhou Institute of Chemical Physics Chinese Academy of Sciences Tianshui Middle Road 18 Lanzhou 730000 P. R. China; ^2^ Department of Heterogeneous Reactions Max‐Planck‐Institut für Chemische Energiekonversion Stiftstraße 34‐36 45470 Mülheim an der Ruhr Germany

**Keywords:** CO_2_ transformation, heteroatom doping, metal‐free catalysis, nanocarbon

## Abstract

Due to high thermal stability, the selective cleavage of the C=O bond in CO_2_, catalyzed by heterogeneous catalysts, is quite challenging, especially in thermal catalytic areas. For the first time, metal‐free active sites for thermal CO_2_ activation and transformation are constructed on the nanocarbon surface in this work. The potential H‐assisted cleavage of the activated C=O bond to produce CO on the metal‐free catalyst is predicted and further confirmed by its reduction using silane as a hydrogen resource. With in situ infrared spectroscopy and theoretical calculations, the evolution process of CO_2_ on the surficial N, B heteroatomic sites of the nanocarbon, providing valuable insights into the reaction mechanism of CO_2_ transformation is revealed. This unprecedented finding not only enhances the understanding of the metal‐free catalytic process for the selective cleavage of the C=O bond in CO_2_ but also provides more opportunities for designing high‐efficiency, low‐cost, and scalable heterogeneous carbon‐based catalysts in thermal CO_2_ transformation processes.

## Graphical abstract

Nitrogen and boron atoms are doped onto the nanocarbon surface to construct an efficient heterogeneous metal‐free catalyst. This catalyst, activate CO_2_ effectively, reducing it to CO with silane through a thermal route. This unprecedented finding sheds light on the application of heterogeneous metal‐free carbon materials in thermal CO_2_ transformation.

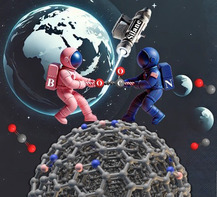

## 1 Introduction

Heterogeneous metal‐free catalysts based on carbon materials have attracted considerable attention and demonstrated promising applications in electrocatalysis (oxygen reduction reaction,^[^ ^1^ ^]^ oxygen evolution reaction,[^1^, ^2^] and CO_2_ reduction^[^ ^3^ ^]^) as well as in thermal catalysis (direct or oxidative dehydrogenation of alkene,^[^ ^4^ ^]^ oxidation,^[^ ^5^ ^]^ hydrogenation,^[^ ^6^ ^]^ dehydration,^[^ ^7^ ^]^ Beckmann rearrangement^[^ ^8^ ^]^). A review of these relevant literature reveals that metal‐free carbon catalysts are primarily active toward O_2_, H_2_O_2_, H_2_O, H_2_, and some small organic molecules, particularly in electrocatalytic applications. In contrast, their use in thermal CO_2_ activation and transformation remains rare, largely due to the inherent stability of CO_2_. Understanding how carbon‐based catalysts interact with various reactants is crucial for elucidating their fundamental catalytic roles, especially given the distinct behavior of carbon catalysts compared to traditional metal‐based systems. This topic has drawn significant interest owing to both its scientific relevance and the urgent need to address CO_2_‐related environmental concerns.^[^ ^9^ ^]^

Carbon materials with a perfect graphitic structure are often considered catalytically inert.^[^ ^10^ ^]^ However, real‐world carbon materials inevitably contain structural imperfections, such as oxygen‐containing functional groups, dangling bonds, topological defects, edge sites, and heteroatoms, that disrupt the uniform electronic structure and localize electrons at reactive sites, thereby endowing them with catalytic activity.^[10b]^ Among these modifications, heteroatom doping has proven to be an effective strategy for tuning both the microstructure and electronic properties of carbon surfaces. This is achieved by leveraging differences in electronegativity, atomic radius, and other factors between the dopants and carbon atoms. Nitrogen and boron, adjacent to carbon in the periodic table, are commonly employed to modify carbon materials,[^1^, ^4^, ^11^] which have shown excellent ability to synergistically adsorb and activate CO_2_ in systems such as boron nitride and frustrated Lewis pairs frameworks.^[^ ^12^ ^]^ These findings suggest strong potential for N, B codoped carbon materials in thermally driven CO_2_ activation and transformation. However, the feasibility and specific mechanisms of CO_2_ activation at N, B codoped sites on carbon surfaces remain poorly understood.

In this work, we propose an effective strategy to incorporate nitrogen and boron atoms onto the nanocarbon surface. The resulting carbon structures, featuring fully exposed N and B heteroatomic sites, are readily scalable and exhibit strong potential for CO_2_ adsorption and activation. We investigate the nature of the active sites and the underlying mechanisms, demonstrating that N–B sites are capable of adsorbing CO_2_ and selectively cleaving the C=O bond with assistance from an activated silane. To the best of our knowledge, this is the first report of a metal‐free, N, B codoped nanocarbon catalyst capable of thermally activating and transforming CO_2_. Furthermore, we elucidate the CO_2_ activation mode at N–B heteroatomic sites on the carbon surface. The insights presented here offer valuable guidance for advancing CO_2_‐related reactions in the field of heterogeneous metal‐free catalysis.

## 2 Results and Discussion

**Figure 1:**
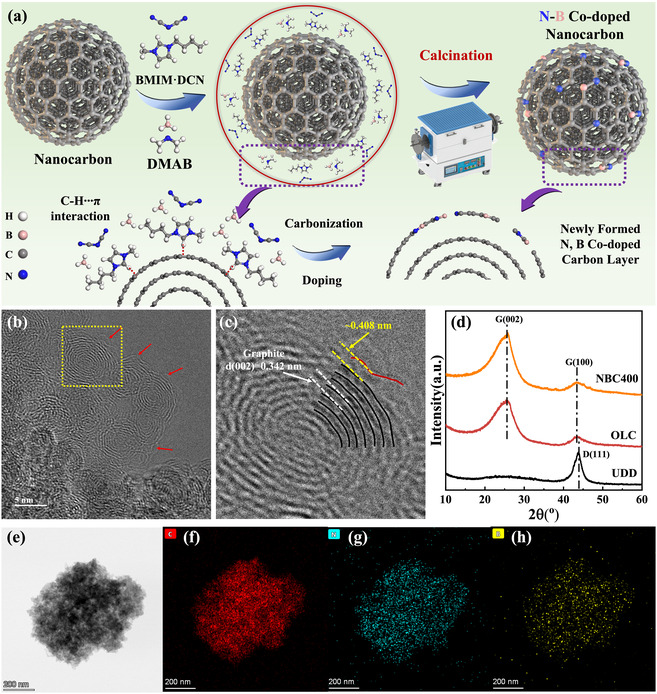
a) Schematic illustration of the formation procedure for N, B codoped nanocarbon; b) HRTEM of NBC400; c) the magnified pattern of the marked area in (b); d) XRD spectra of samples; e) region selected for EDX mapping of NBC400; f) EDX mapping of C atoms; g) EDX mapping of N atoms; and h) EDX mapping of B atoms.

In this work, we present an efficient and scalable strategy to construct a nitrogen–boron codoped structure on a model graphitic carbon surface, specifically, onion‐like carbon (OLC), as illustrated in **Figure** **1**a. OLC was synthesized by annealing ultradispersed nanodiamonds at 1500 °C for 4 h under an argon atmosphere (Figure S1, Supporting Information). Comparative analysis of the Fourier transform infrared (FTIR) spectra, X‐ray photoelectron spectroscopy (XPS), and elemental composition (Figure S2, S3, and Table S1, Supporting Information) confirms the near‐complete removal of oxygen‐containing functional groups and the formation of a highly graphitized carbon surface during the synthesis of OLC. To introduce N and B dopants, 1‐butyl‐3‐methylimidazolium dicyanamide and borane were employed as the respective precursors. The imidazolium‐based ionic liquid (IL) interacts with the graphitic surface via CH···π interactions, leading to the formation of a uniform, thin IL layer on the OLC surface.^[^ ^6^, ^13^ ^]^ Subsequent calcination under vacuum in the presence of borane enables the graphitic surface to trap decomposed debris, resulting in the formation of a new N, B codoped carbonaceous layer on the nanocarbon surface. The resulting materials are denoted as NBC*x*, where *x* represents the annealing temperature. Their surface area and pore volume are summarized in Figure S4 and Table S1, Supporting Information. The morphology of NBC400 was examined via high‐resolution transmission electron microscopy (HRTEM), as shown in Figure 1b. NBC400 exhibits multilayered *sp* ^2^ carbon shells with an interlayer spacing of 0.342 nm, characteristic of the (002) lattice of graphitic carbon, consistent with X‐ray diffraction (XRD) results (Figure 1d). A newly formed, highly defective N, B codoped carbon monolayer, indicated by a red arrow in Figure 1c, exhibits a slightly expanded spacing of ≈0.408 nm at the outermost layer. Elemental mapping by energy‐dispersive X‐ray spectroscopy (EDX) (Figure 1e–h) shows a uniform distribution of nitrogen and boron across the surface, confirming the successful codoping of the OLC.^[13c]^

**Figure 2:**
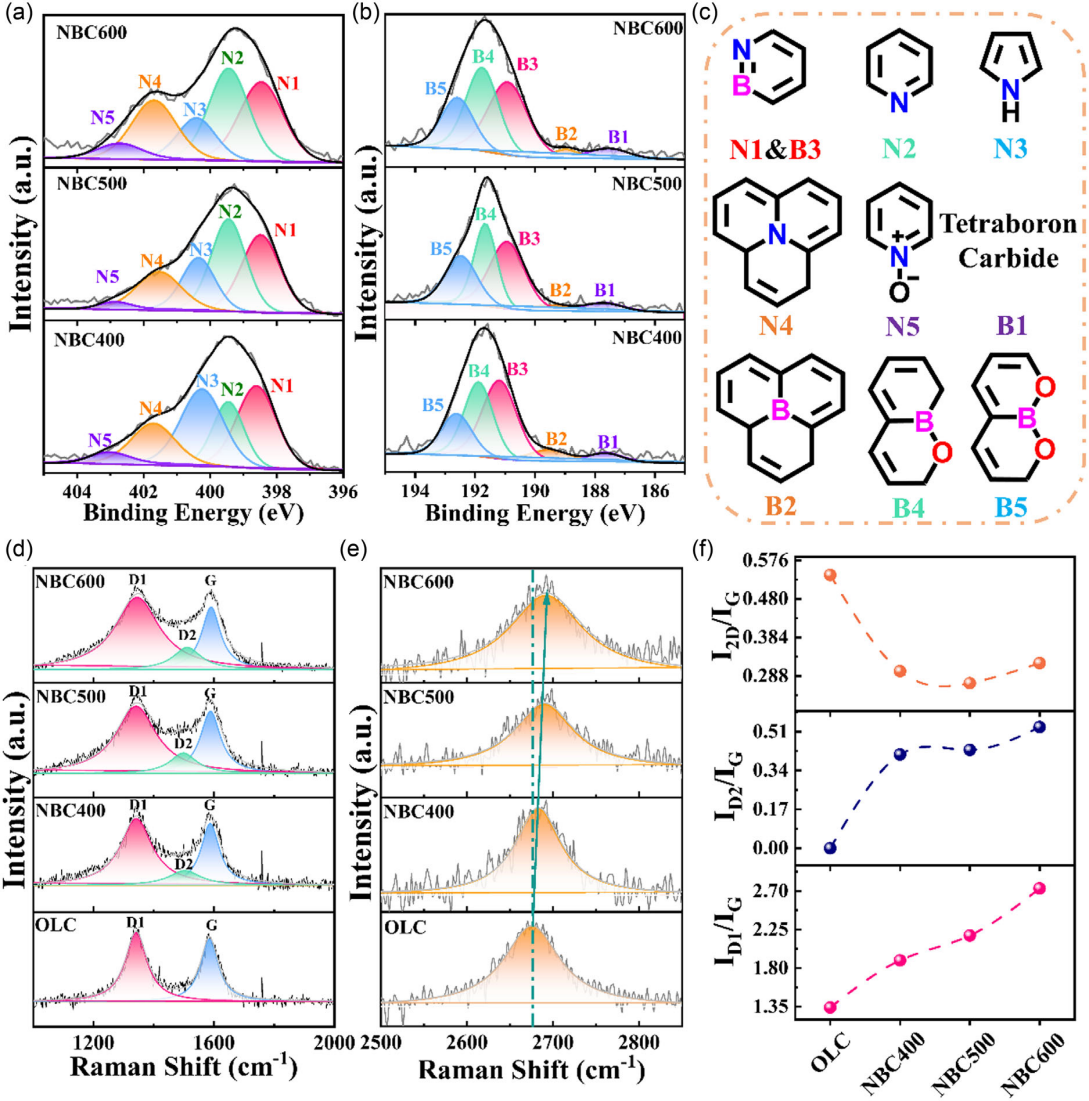
a) The deconvoluted N 1*s* XPS spectra of NBC*x* samples; b) the deconvoluted B 1*s* XPS spectra of NBC*x* samples; c) the possible structure of different N or B species; d) the deconvoluted D1, D2, and G bands in Raman spectra of OLC and NBC*x* samples; e) the 2D band in Raman spectra of OLC and NBC*x* samples; and f) *I*_D1_/*I*_G_, *I*_D2_/*I*_G_, and *I*_2D_/*I*_G_ of OLC and NBC*x* samples.

XPS of different samples was collected to investigate the surface composition, nitrogen, and boron species. The pristine OLC has 99 at% C atoms and only a small amount of O atoms, but no N or B atoms, as shown in Figure S3 and Table S1, Supporting Information. In addition, trace metal impurities—such as Fe, Cu, and Al—were barely detectable in the catalysts, with concentrations remaining at or below the parts‐per‐million (ppm) level.^[^ ^14^ ^]^ After the introduction of N and B, all NBC*x* samples exhibit significant N and B XPS signals, suggesting the efficiency of the N, B codoping method proposed by this work. The C 1*s* spectra of both OLC and N, B codoped carbon samples are dominated by signals attributed to *sp* ^2^‐hybridized carbon and the characteristic π–π* satellite peak, as shown in Figure S3b, Supporting Information. However, the spectral differences between the pristine OLC and its N, B codoped counterpart are insufficient to provide meaningful insight into the doping structure, primarily due to the significant contribution from the OLC core. Therefore, to better elucidate the nature of nitrogen and boron incorporation, high‐resolution N 1*s* and B 1*s* spectra were further analyzed. The N 1*s* spectra of all the three N, B codoped samples could be deconvoluted into N–B species (N1), pyridinic N (N2), pyrrolic N (N3), graphitic N (N4), and pyridinic N oxide (N5), whose possible structure is proposed, as shown in **Figure** **2**a,c. N–B species (B3) could also be found in the B 1*s* spectra with B_4_C (B1), BC_3_ (B2), BC_2_O (B4), and BCO_2_ (B5) in the deconvolution of B 1*s* spectra, whose possible structure is depicted in Figure 2c.[^1^, ^11^, ^15^] The N and B contents of the samples derived from XPS analysis are summarized in Table S1, Supporting Information (in the supporting information). The nitrogen content decreases with increasing preparation temperature, which can be attributed to the release of nitrogen species in the form of NH_3_, HCN, and N_2_.^[^ ^16^ ^]^ Meanwhile, the percentage of pyrrolic N decreases while the percentage of pyridinic N and graphitic N increases, suggesting the formation of a more graphitic lattice with six‐membered rings from surface carbonaceous debris with increasing temperature (Figure S6, Supporting Information). This also result in a slight decrease in N–B species content (1.9% for NBC400, 1.6% for NBC500, and 1.3% for NBC600). On the contrary, the B content almost remains steady at moderate temperatures (400–600 °C) likely due to the strong C—B bonds and the low volatility of boron species. It may even exhibit a slight increase, possibly due to the concurrent loss of nitrogen and carbon. Ultraviolet photoelectron spectroscopy was further used to investigate the valence shell electronic structure of NBC*x* samples, as shown in Figure S7, Supporting Information. The 2*p* π peak of OLC and NBC*x* samples is indicative of their graphitic structure. While the less prominent 2*p* π peaks and the steeper increase in density of states in the region of 3–7 eV for NBC*x* samples compared with OLC could be attributed to B‐ and N‐doping.[^1^, ^14^, ^17^] Moreover, NBC*x* samples have a larger work function than OLC, and NBC400 exhibits the lowest work function among NBC*x* samples, suggesting that the minimum energy is required for the inner electrons to escape from their nuclei on the NBC400 surface.

Raman spectroscopy is a powerful technique for studying the surface structure and composition of graphitic, disordered, and amorphous carbon materials.[^1^, ^18^] The visible spectra using a 532 nm laser source could give more information about defects.^[^ ^19^ ^]^ From Figure 2d,e, it could be found that the pristine OLC possesses the D1 band, G band, and 2D band at 1350, 1580, and 2660 cm^−1^, respectively, which is in agreement with the previous report.[^1^, ^10^] The G band is attributed to the in‐plane bond‐stretching motion of *sp* ^2^ hybridized C atom pairs of E_2g_ symmetry from the graphitic rings or chains.^[18c,d]^ The D1 band is attributed to the result from the graphitic ring breathing mode of A_1g_ symmetry involving phonons near the K point at the Brillouin zone boundary, which is closely related to the graphitic lattice and could only be observed in the presence of defects near the graphitic lattice. The 2D band is assigned to a second‐order process related to a phonon near the K point, activated by double resonance processes, and could serve as a very sensitive probe for characterizing specific *sp* ^2^ nanocarbons.^[^ ^19^, ^20^ ^]^ Upon the introduction of nitrogen and boron, the relative intensity ratio of the D1 band to the G band (*I*_D1_/*I*_G_), calculated from the integrated peak areas, increases from 1.34 for OLC to 1.89 for NBC400, as shown in Figure 2f. This increase is likely attributed to the formation of new carbonaceous structures on the surface of OLC.^[^ ^6^, ^13^, ^21^ ^]^ The symmetry breaking caused by the heteroatoms doping in the carbon skeleton is another incentive for the increase in D1 band intensity.^[18e]^ The width of the D1 band is also increased from the full width at half maximum (FWHM) of OLC (88 cm^−1^) to FWHM of NBC400 (130 cm^−1^), indicating that the newly generated N, B codoped carbon structure lacks the perfect fullerene‐like structure. *I*_D1_/*I*_G_ increases with the preparation temperature, which could be attributed to the formation of a more graphitic lattice from the thermal coupling of carbonaceous debris with increasing temperature.[^18^, ^19^] This result is consistent with the graphitization of carbonaceous debris attached to the OLC surface from the XPS analysis. A D2 band was also observed from the deconvolution of the Raman spectra of NBC*x*, which is assigned to the amorphous carbon.[^1^, ^10^, ^13^] These results all suggest that the newly formed layer on the OLC surface is composed of a large volume of graphitic lattice with defects and amorphous carbon. Compared with the pristine OLC, the 2D band intensity and *I*_2D_/*I*_G_ of NBC*x* all decrease, indicating that the N, B codoped carbonaceous structure would conceal the stacking order of the fullerene‐like layers of OLC. Also, the 2D band position upshift for NBC*x* compared to OLC is observed from Figure 2e, indicating the successful insertion of heteroatoms into the carbon surface, consistent with the fully resonant process in the Raman theory.^[^ ^22^ ^]^

The presence of N, B codoped carbon structures on the surface of the NBC_*x*_ samples was confirmed by the aforementioned characterizations. To evaluate the impact of N, B codoping on CO_2_ adsorption performance, CO_2_ physisorption experiments were conducted. The corresponding CO_2_ adsorption–desorption isotherms are presented in Figure S4c, Supporting Information. Notably, all NBC*x* samples exhibit higher CO_2_ uptake compared to OLC, despite OLC possessing the highest surface area and pore volume. Interestingly, the desorption branches of the CO_2_ isotherms for all NBC*x* samples are clearly located above their corresponding adsorption branches, whereas the adsorption and desorption curves for OLC are nearly superimposed. This hysteresis suggests the presence of relatively strong interactions between CO_2_ and the NBC*x* samples. Given the comparable pore structures of OLC and NBC*x* samples below 10 nm (Figure S4b, Supporting Information), the enhanced CO_2_ affinity is likely attributed to the N, B codoped surface functionalities rather than physical textural differences.

**Figure 3:**
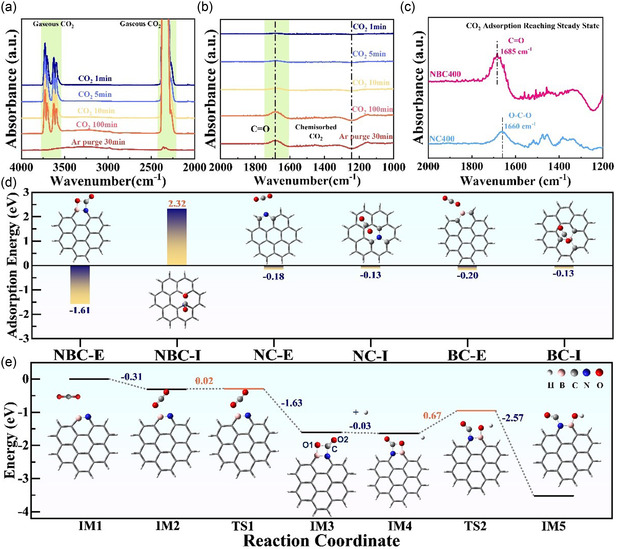
The in situ FTIR spectra of CO_2_ adsorption on NBC400 during CO_2_ blowing and argon purging at wavenumber of a) 4000–2000 cm^−1^ and b) 2000–1000 cm^−1^; c) the in situ FTIR spectra of CO_2_ adsorption on NC400 compared with NBC400 after reaching steady state; d) the optimized adsorption structure and energy of CO_2_ on different models including NBC‐E, NBC‐I, NC‐E, NC‐I, BC‐E, and BC‐I; and e) the reaction path of CO_2_ adsorption, activation, and reaction with H.

The chemical adsorption and activation capability of NBC400 for CO_2_ was further investigated using in situ FTIR spectroscopy. As shown in **Figure** **3**a,b, characteristic signals of gaseous or physically adsorbed CO_2_ emerged after just 1 min of CO_2_ exposure, appearing prominently in the regions of 2400–2300 and 3750–3550 cm^−1^. These bands correspond to the ν_3_ antisymmetric stretching vibration, and the ν_3_ + 2ν_2_ and ν_1_ + ν_3_ combination modes of CO_2_, respectively.[^12^, ^23^] Notably, significant changes were observed in the 1800–1000 cm^−1^ region with increasing CO_2_ exposure time, which is commonly associated with chemisorbed CO_2_ species.[^12^, ^23^, ^24^] A distinct peak appeared at ≈1685 cm^−1^, growing in intensity over time until reaching a plateau. This peak is attributed to the C=O stretching vibrations (ν_C=O_) of chemisorbed CO_2_, closely resembling activated CO_2_ species reported for frustrated P–B Lewis pairs (1692–1700 cm^−1^) and frustrated N–B Lewis pairs (1707 cm^−1^).^[^ ^25^ ^]^ Concurrently, a decrease in absorbance within the 1380–1150 cm^−1^ range was detected, likely due to interactions between chemisorbed CO_2_ and the NBC400 surface.^[^ ^6^, ^26^ ^]^ After CO_2_ introduction ceased and the system was purged with Ar, the signals corresponding to gaseous CO_2_ disappeared. Remarkably, the chemisorbed CO_2_ signals within the 1800–1000 cm^−1^ region remained almost unchanged, indicating a strong and persistent interaction between CO_2_ and the N, B codoped nanocarbon surface, a phenomenon rarely reported in previous studies.

As is well known, nitrogen (N) doping can introduce adsorption sites for CO_2_.^[^ ^27^ ^]^ To investigate the origin of the CO_2_ adsorption capacity in NBC*x*, in situ FTIR spectra of CO_2_ adsorption on N‐doped NC400 were collected for comparison. As shown in Figure 3c, a clear difference is observed between the FTIR signals of CO_2_ adsorption on NC400 and NBC400. Notably, the FTIR peak for CO_2_ adsorption on NC400 appears at ≈1660 cm^−1^, corresponding to asymmetric O—C—O stretching vibrations. This differs from the FTIR signal at around 1685 cm^−1^ observed for NBC400.[^13^, ^28^] These results suggest that the CO_2_ adsorption mode on N‐doped nanocarbon is distinct from that on N, B codoped nanocarbon. Although the codoped nanocarbon also exhibits some basicity, as indicated by in situ pyrrole adsorption FTIR results in Figure S9, Supporting Information, the adsorption mechanisms differ. Physical CO_2_ adsorption measurements further support this conclusion. NC400 shows a lower CO_2_ adsorption capacity (0.26 mmol) compared to NBC400 (0.37 mmol), despite having a slightly larger surface area (230.7 m^2^ g^−1^ vs. 217.7 m^2^ g^−1^), as shown in Figure S5, Supporting Information. These findings indicate that basic sites alone do not account for the enhanced CO_2_ adsorption in NBC*x*; rather, the synergistic effect of N and B codoping plays a critical role.

To further explore this synergy, DFT calculations were conducted to investigate CO_2_ adsorption on various heteroatomic sites. Based on characterization results and previous studies, carbon models with isolated N‐doping, B‐doping, and N, B codoping in different configurations were constructed and optimized, as shown in Figure S10, Supporting Information.^[^ ^6^, ^29^ ^]^ CO_2_ adsorption on these models was analyzed to identify potential active sites. The optimized adsorption configurations and corresponding adsorption energies are presented in Figure 3d. Among the models, CO_2_ adsorption on the N, B codoped model with an edge‐located N–B site (NBC‐E) is the most stable. In contrast, CO_2_ adsorption on the interior N–B site model (NBC‐I) is unstable, as indicated by its positive adsorption energy. N, B codoping at the edge induces the distortion in the graphitic carbon structure with the formation of N—B bond as shown in Figure S11, Supporting Information. Molecular orbital analysis reveals that the p electrons contribute to the N–B bond in the highest occupied molecular orbital (HOMO), which is accessible and favorable for interaction with CO_2_ molecules. Therefore, the N–B site at the edge is identified as a potential CO_2_ adsorption site. The adsorption‐activation process was further investigated using the NBC‐E model, as shown in Figure 3e.

As shown in Figure 3e, CO_2_ is initially absorbed physically near the N–B site, with an adsorption energy of −0.31 eV. In this intermediate state (**IM2**), the oxygen atom is positioned close to the boron atom, while the CO_2_ molecule remains nearly linear and intact. Upon overcoming a minor energy barrier of 0.02 eV via a transition state (**TS1**), CO_2_ forms a more stable adduct (**IM3**) with the NBC‐E site, characterized by a significantly stronger adsorption energy of −1.61 eV. In **TS1**, the O–C–O angle of the CO_2_ molecule is slightly distorted to 169°, and the vibrational mode corresponding to the sole imaginary frequency indicates the transition toward **IM3** (Figure S12b, Supporting Information). In the resulting **IM3** adduct, CO_2_ forms covalent bonds with the N and B atoms, and the O–C–O angle is substantially reduced to 130° with strong molecular distortion. Natural Bond Orbital (NBO) analysis shows a bond population of 1.97e for the O1–B bond, confirming its covalent nature. The C–O1 bond length increases from 1.16 to 1.42 Å, with a bond population of 1.98e, characteristic of a single bond, suggesting activation of the CO_2_ molecule. The most intense vibrational mode in **IM3** (Figure S12c, Supporting Information) corresponds to a C=O stretching vibration, consistent with the observed IR peak at 1685 cm^−1^ in Figure 3b. Moreover, the desorption of CO_2_ from the adduct would require overcoming a high backward energy barrier of 1.32 eV, making desorption difficult. This aligns with the nearly unchanged FTIR spectra in the 2000–1000 cm^−1^ range, further confirming that the edge‐located N–B sites serve as effective active sites for CO_2_ activation.

**Figure 4:**
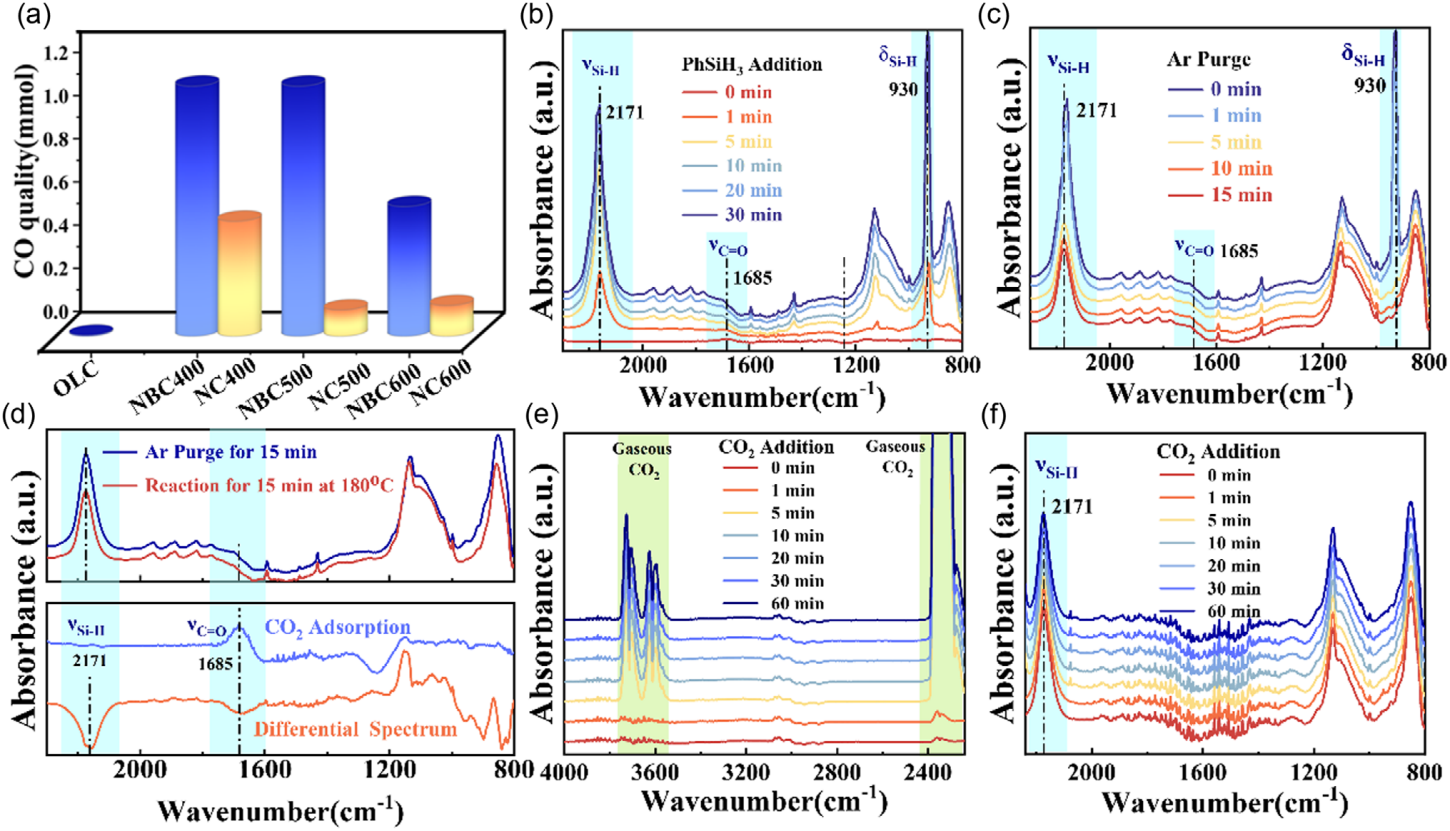
a) Catalytic performance of various carbon‐based catalysts (reaction condition: 10 mg catalyst, 1 mL dodecane, 2 mmol PhSiH_3_, 4 MPa CO_2_, 180 °C, 16 h); b) in situ FTIR spectra of PhSiH_3_ addition into CO_2_ adsorbed NBC400 at wavenumber of 2240–800 cm^−1^; c) in situ FTIR spectra of Ar purge after PhSiH_3_ addition into CO_2_ adsorbed NBC400 at wavenumber of 2240–800 cm^−1^; d) in situ FTIR spectra of the reaction at 180 °C for 15 min and the differential spectrum; and in situ FTIR spectra of CO_2_ addition after PhSiH_3_ addition on fresh NBC400 reaching the steady state at the wavenumber of e) 4000–2240 cm^−1^ and f) 2240–800 cm^−1^.

Following activation, the reductive transformation of CO_2_ becomes critical for practical applications. Selective cleavage of the C=O bond to produce CO is of great significance in academia and industry, as CO is a key industrial chemical.^[^ ^30^ ^]^ To explore the possibility of this transformation, an activated hydrogen atom was introduced to the **IM3** adduct, and theoretical calculations were performed. As shown in Figure 3e, hydrogen first interacts physically with the **IM3** adduct, then facilitates cleavage of the activated C—O bond via transition state **TS2**, with an activation barrier of 0.67 eV. Motivated by this theoretical insight, PhSiH_3_ was used as a reductant to experimentally assess CO_2_ reduction on NBC*x* samples. The catalytic performance is summarized in **Figure** **4**a and **Table** **1**. CO is the primary reduction product of CO_2_, with the oxygen atom from CO_2_ being captured by PhSiH_3_ to form siloxane as a byproduct.^[^ ^31^ ^]^ At elevated reaction temperatures, the siloxane undergoes further transformation into condensed silicone species, as confirmed by the ^29^Si nuclear magnetic resonance spectrum of the liquid product shown in Figure S14, Supporting Information. No CO production was observed in the absence of either the catalyst or the reductant. The pristine OLC also showed negligible catalytic activity, whereas NBC400 exhibited excellent catalytic performance. NBC500 and NBC600 also demonstrated good activity, though slightly reduced, likely due to lower N–B species content, fewer edge‐located N–B sites, or a higher work function.^[^ ^32^ ^]^ To emphasize the cooperative effect of N and B codoping, nitrogen‐doped NC*x* samples (without B) were synthesized and tested under identical reaction conditions. These solely N‐doped materials showed significantly lower catalytic activity than their NBC*x* counterparts synthesized at the same temperatures, highlighting the critical role of N, B codoping in both CO_2_ activation and transformation. As shown in Table 1, the CO yield catalyzed by NBC400 increased with reaction time. Higher reaction temperatures also promoted greater CO_2_ reduction. Increasing the amount of reductant led to higher CO production, reinforcing the catalytic efficiency of NBC*x* samples. Additionally, the recyclability of NBC400 was tested (Figure S13, Supporting Information), revealing that its CO yield remained nearly unchanged even after five cycles, indicating strong catalyst stability.

**Table 1:** Catalytic performance of NBC400 under different reaction conditions. The main variations in the parameter are highlighted in bold.

Entrya)	Catalyst	PhSiH_3_ [mmol]	Temperature [°C]	Time [h]	CO yield [mmol]
1	**No**	2	180	16	0
2	NBC400	**No**	180	16	0
3	NBC400	2	180	**4**	0.195
4	NBC400	2	180	**8**	0.655
5	NBC400	2	180	**12**	1.176
6	NBC400	**2**	**180**	**16**	1.479
7	NBC400	2	**140**	16	0.289
8	NBC400	2	**160**	16	1.157
9	NBC400	2	**200**	16	2.312
10	NBC400	**1**	180	16	0.686
11	NBC400	**4**	180	16	2.231

a) Reaction condition: 10 mg catalyst, 1 mL dodecane as solvent, 4 MPa CO_2_, stirring speed of 800 rpm.

To investigate the reaction pathway and mechanism, in situ FTIR spectroscopy was employed to monitor the reduction of activated CO_2_ by PhSiH_3_. Initially, the in situ FTIR spectra of CO_2_ adsorption remained nearly unchanged under Ar purging, as shown in Figure 3b. PhSiH_3_ was then introduced via Ar passing through a bubbler containing PhSiH_3_. The moment Ar began flowing through the bubbler was designated as the starting point, and spectra were recorded at regular intervals (Figure 4b). Characteristic IR peaks of PhSiH_3_ at 930 and 2171 cm^−1^ increased with bubbling time. After reaching a steady state, physically adsorbed PhSiH_3_ was removed by Ar purging, and subsequent FTIR spectra were collected (Figure 4c). It could be found that the IR peak intensity of ν_Si–H_ decreased gradually and finally reached a steady state, while the IR peak intensity of δ_Si–H_, unique to the PhSiH_3_ molecule, decreased until it disappeared almost completely. These results suggest that PhSiH_3_ could be adsorbed onto NBC*x* samples chemically and be activated effectively.

A decrease in the IR peak at 1685 cm^−1^ (ν_C=O_) following PhSiH_3_ introduction seemingly suggested possible CO_2_ reduction in Figure 4b. However, further in situ FTIR experiments revealed that this decrease was just due to the influence of PhSiH_3_ IR peaks. Specifically, PhSiH_3_ was first introduced into the in situ diffuse reflectance infrared fourier transform (DRIFT) cell containing fresh NBC400 and purged by Ar until the spectra reached a steady (Figure S14, Supporting Information). CO_2_ was then introduced, and although strong signals of gaseous CO_2_ appeared in the regions of 2400–2300 and 3750–3550 cm^−1^, the IR peaks attributed to PhSiH_3_ remained essentially unchanged (Figure 4e,f). This indicates a strong interaction between PhSiH_3_ and NBC400 and suggests that, at 40 °C, the reaction between CO_2_ and PhSiH_3_ is minimal, further corroborated by the low CO yield of only 0.0002 mmol under these conditions.

Subsequently, the in situ DRIFT cell, as in Figure 4c, was sealed and heated to 180 °C to promote the reduction of activated CO_2_ by chemisorbed PhSiH_3_. FTIR spectra were collected after 5 min of reaction at 180 °C, and a differential spectrum was obtained by subtracting the prereaction spectrum in Figure 4d. In this differential spectrum, downward peaks at 1685 cm^−1^ (ν_C=O_) and 2171 cm^−1^ (ν_Si–H_) indicate the consumption of both C=O and Si–H groups, confirming their participation in the reaction. The gas product was collected from the in situ FTIR cell and analyzed by gas chromatography, which confirmed CO as the sole reduction product consistent with catalytic activity results. In brief, the activated CO_2_ by N, B heteroatomic sites could be reduced by Si–H of the chemisorbed PhSiH_3_, where N, B heteroatomic sites serve as catalytic sites to promote the cleavage of the C=O bond and release CO as the sole product.

## 3 Conclusion

In summary, we have successfully synthesized N, B codoped nanocarbon materials featuring highly active N–B sites. CO_2_ physisorption measurements indicate a specific interaction between CO_2_ and the NBC*x* samples. Combined in situ FTIR analysis and DFT calculations further demonstrate that NBC*x* samples exhibit strong chemisorption capacity for CO_2_ and enable its activation, evidenced by C=O bond elongation and O–C–O angle distortion. The theoretically predicted selective cleavage of the C=O bond by hydrogen is experimentally validated through the reduction of CO_2_ to CO using PhSiH_3_ as a reductant on NBC*x* samples. In situ FTIR studies elucidate the reaction mechanism, revealing that the activated CO_2_ is reduced to CO via C=O bond cleavage facilitated by the Si–H of chemisorbed and activated PhSiH_3_. To the best of our knowledge, this study represents the first report of metal‐free N, B codoped nanocarbon materials applied in thermal catalytic CO_2_ transformation. Moreover, it uncovers the intrinsic activation mechanism of CO_2_ over these materials. These findings not only offer a new approach to thermal CO_2_ activation but also broaden the application potential of metal‐free nanocarbon materials as catalysts and catalytic supports.

## Conflict of Interest

The authors declare no conflict of interest.

## Supporting information

Supplementary Material

## Data Availability

The data that support the findings of this study are available from the corresponding author upon reasonable request.
