# Supplementary material for: Heteroatomic Sites on Carbon: Thermal CO2 Activation and Transformation by Metal‐Free Catalysis
Source: ChemSusChem. 2025 Aug 20;18(18):e202501052. doi: 10.1002/cssc.202501052 (PMC12456386; doi:10.1002/cssc.202501052)
Supplement: Supplementary file 1 — Supplementary Material [file CSSC-18-e202501052-s001.pdf]

Supporting Information

Heteroatomic Sites on Carbon: Thermal CO_2 Activation and Transformation by Metal-free Catalysis

Peng Zhang,[a,b] Chaoan Liang, [a] Tao Du,[a] Jiali Sun, [a] Bohao Pang, [a] Saskia Heumann, *[b] Yuxiao Ding*[a]

Dedication ((optional))

[a] Dr. P. Zhang, Mr. T. Du, Mr. C. Liang, Dr. J. Sun, Mr. B. Pang, Prof. Dr. Y. Ding,
State Key Laboratory of Low Carbon Catalysis and Carbon Dioxide Utilization
Lanzhou Institute of Chemical Physics, Chinese Academy of Sciences
Tianshui Middle Road 18, 730000, Lanzhou, P.R. China
E-mail: yuxiaoding@licp.cas.cn

[b] Dr. P. Zhang, Dr. S. Heumann
Max-Planck-Institut für Chemische Energiekonversion
Stiftstraße 34-36, 45470 Mülheim an der Ruhr (Germany)
E-mail: Saskia.Heumann@cec.mpg.de

Table of Contents

1. Experimental Section 2

2. Supporting data and discussion 5

References.. 18

1. Experimental Section

***Reagents*:** Purified ultra-dispersed nanodiamonds (UDD) were purchased from Beijing Grish Hitech Co., Ltd, prepared through detonation method and followed by acid washing to remove amorphous carbon and metal impurity. 1-butyl-3-methylimidazolium dicyanamide (BMIM·DCN, 98%) was provided by Shanghai Chengjie Chemical Co., Ltd. Dimethylamino borane (DMAB, 98%), dodecane (98%) and phenyl silane ($PhSiH_3$, 97%) were purchased from Energy Chemical Co., Ltd. Ultra-pure Ar and CO_2 gas were provided by Air Liquide.

***Preparation of N, B co-doped carbonaceous catalysts*:** UDD was first annealed under Ar atmosphere at 1500°C for 4h to obtain onion-like carbons (OLC), which have almost perfect curved graphitic carbon structure. OLC is selected as the starting material to prepare N, B co-doped carbonaceous catalysts to avoid the interference from carbon substances. [1] BMIM·DCN and DMAB are utilized as nitrogen and boron sources respectively and the synthesis procedure is described as follows. BMIM·DCN was first supported on OLC with equal weight through impregnation in alcoholic solution and the following complete desiccation. Then DMAB with the same weight as OLC was loaded at the bottom of a graphite crucible and covered with the BMIM·DCN/OLC obtained above. The whole graphite crucible was transferred into the central region of a tubular furnace, which was vacuumed and exchanged with Ar three times to remove oxygen and moisture as far as possible. The whole tubular furnace was then sealed and stayed vacuumed. The temperature was then increased from room temperature to setup temperature with the rate of 10°C min^{-1} and remained for 1h. Three N, B co-doped carbonaceous catalysts were prepared at 400°C, 500°C and 600°C, denoted as NBC400, NBC500 and NBC600, respectively.

To underscore the importance of N, B co-doping on catalytic performance, N doped carbonaceous catalysts were also prepared as reference similar to the method described above except that DMAB was absent. N doped carbonaceous catalysts were prepared at 400°C, 500°C and 600°C, denoted as NC400, NC500 and NC600, respectively.

***Characterization*:** Microscopic morphology of N, B co-doped OLC was investigated using the high-resolution transmission electron microscope (HRTEM, Talos F200X, Thermo Scientific). The sample was first deposited onto carbon film copper grids before TEM analysis. EDX mapping was also collected to study the elemental distribution with the help of TEM. The fine structure was further studied on the Titan Themis G3 ETEM (Thermo Scientific Company), which was used at 300 kV with a Cs corrector for parallel imaging (CEOS GmbH) and a measured resolution better than 1.0 Å.

XRD spectra of samples was collected in X-ray diffractometer (SmartLab 3KW, Rigaku Corporation).

Raman spectra of samples was collected on laser confocal micro-Raman spectrometer (Thermo Scientific Company, DXR) with the 532 nm laser (Cobolt), whose power was kept about 1 mW with exposure time of 4 min.

XPS spectra of samples were collected on Thermo Scientific NEXSA Surface Analysis system with a monochromatized Al Kα X-ray source (hv=1486.6 eV) and the power of 150 W.

N_2 and CO_2 physical adsorption on NBC*x* samples were performed on the physical adsorption instrument (Micromeritics 3Flex). The samples were first pretreated under vacuum at 473 K for 6 h to remove physically adsorbed gases. Nitrogen physical adsorption was then carried out at 77 K at the relative pressure range of 0~1. The specific surface area of samples was calculated using the Brunauer-Emmett-Teller (BET) model and the pore size distribution of samples was derived using the Barrett-Joyner-Halenda (BJH) model. Physical CO_2 adsorption was carried out at 298 K at the pressure range of 0~760 mm Hg.

In-situ FTIR on carbon-based catalysts was performed on Nicolet iS50 spectrometer (Thermo Scientific) equipped with a diffuse reflection accessory consisting of a heat chamber, DRIFT cell and MCT-A detector. All spectra were collected over 64 scans at 4 cm^{-1} resolution under desired conditions. In a typical process, the powder sample was first placed in the DRIFT cell and pretreated at 300^oC for 30 min in Ar flow (30 $mL\cdot min^{-1}$) then cooling to 40^oC. And the spectrum of sample was collected as background in the following experiments. CO_2 of 30 $mL\cdot min^{-1}$ was introduced into the DRIFT cell and the spectra were recorded between certain time until the spectra reach steady. Then CO_2 was shut down and purged by Ar until the signals of gaseous CO_2 disappeared. When the spectra remain almost unchanged, $PhSiH_3$ was introduced through Ar running through a bubbler containing $PhSiH_3$. The time Ar starts to enter the bubbler is regarded as the starting point and spectra was collected between certain times. The physically adsorbed or gaseous $PhSiH_3$ was then blown

away through Ar purging until the IR spectra reached steady. The in-situ FTIR cell was sealed, and the temperature was increased to 180°C for reaction. The in-situ FTIR of $PhSiH_3$ addition first and subsequent CO_2 introduction was performed according to the similar procedure described as above.

***Catalytic test*:** All catalytic experiments concerning catalytic reduction of CO_2 to CO by NBC were performed in a high-pressure stainless autoclave equipped with temperature controller and magnetic stirring. A specific catalytic test procedure is described as follows: a certain amount of carbon-based catalyst, dodecane as solvent, and $PhSiH_3$ as reductant are loaded into the autoclave. Air in the sealed autoclave is replaced by 2MPa CO_2 three times and CO_2 of 4MPa is finally charged. The amount of charged CO_2 is determined by subtracting the weight of autoclave before CO_2 charging from that after CO_2 charging.[2] The autoclave is then placed in the heating jacket which has reached the setup temperature and the moment when stirring of 800rpm start is considered as reaction time of zero. After reaction of specific time, the autoclave is removed from the heating jacket and placed into cold water bath.

The gas product is collected in a gasbag and analyzed using gas chromatography (GC9720 plus, Fuli) equipped with two FID detectors and one TCD detector. Low-concentration CO would be converted into CH_4 through methanation and detected by FID detector. The liquid product is collected and analyzed using gas chromatograph mass spectrometer (GCMS-QP2010 SE, Shimadzu), ^{1}H and ^{13}C NMR (AVANCE NEO 400MHZ, Bruker), while there is no liquid product from CO_2 detected.

***Theoretical calculations*:** Theoretical calculations based on density functional theory were performed to investigate the electronic structure, interaction process, catalysis mechanism of CO_2 activation and transformation on N, B co-doped carbonaceous catalysts. Structure optimizations and frequency analysis were all conducted on Gaussian 16 using B3LYP hybrid functional and 6-31+G (d, p) basis set plus dispersion correction to include long range noncovalent interaction,[3] which has been proved efficient in optimization for main group elements.[4] The high precision energy calculations were then performed using PWPB95 hybrid functional and def2-TZVPP basis set on ORCA 4.1.1,[5] where the input file was created with the help of Multiwfn.[6] For systems concerning weak interactions, basis set superposition error (BSSE) correction was conducted for the calculation of interaction energy based on the counterpoise correction method.[7] And the interaction energy of the system could be calculated by the following equation:

$$E_{ads} = E_{A+B}-E_A-E_B+BSSE$$

where A represents carbonaceous catalyst model, B represents reactant, intermediate or product.

2. Supporting data and discussion

The structure of UDD and OLC

The HRTEM images of ultra-dispersed nanodiamonds (UDD) and onion-like carbons (OLC) were collected to illustrate their structure as shown in **Figure S1 a) and b)**. It could be found that UDD mainly has the core of diamond phase with the d (111) spacing of 0.208 nm and amorphous part attached at the surface. After the annealing treatment under Ar atmosphere at 1500 ^{o}C for 4 h, UDD has been transformed into OLC completely with the morphology of sp^2 fullerene-like multilayers and the d (002) spacing of 0.304 nm. The phase change could also be confirmed by XRD spectra in **Figure 1 c)** where UDD has a typical peak at 43.8^{o} from (111) plane of diamond and OLC has the typical peaks at 25.2^{o} and 43.5^{o} from (002) and (100) planes of graphite.

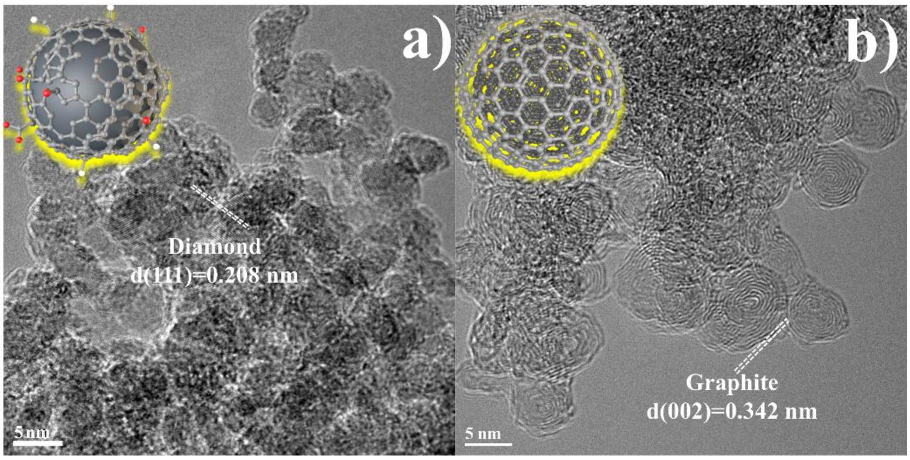

Figure S1 a) HRTEM of UDD; **b)** HRTEM of OLC.

There exist IR peaks for UDD at 1765 cm^{-1} ($v_{C=O}$ from lactones, anhydride, ketone or carboxyl), 1630 cm^{-1}(adsorbed H_2O), 1261 cm^{-1} and 1105 cm^{-1} ($v_{C\text{-}O\text{-}C}$ and $v_{C\text{-}O}$), suggesting the possession of oxygen functional groups in **Figure S2**. While there are no significant IR peaks in the FTIR spectra of OLC and there appear rather strong C 1s signal coupled with weak O 1s signal in the XPS survey for OLC in **Figure S3**. These results indicate that oxygen functional groups have been eliminated almost completely during UDD was transformed into sp^2 hybridized OLC which was utilized as the starting nanocarbon material.

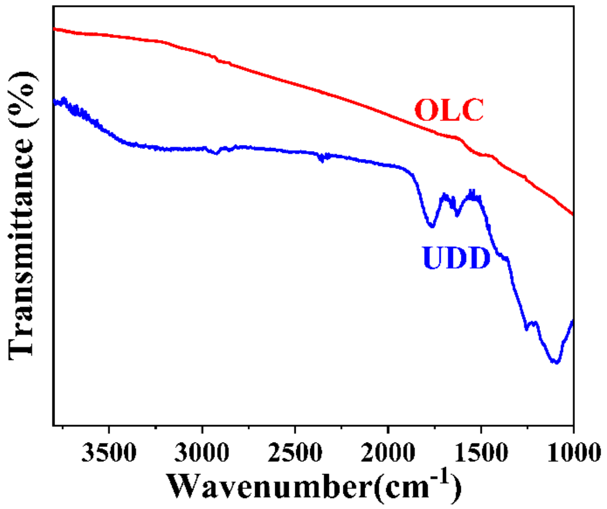

Figure S2. FTIR of UDD and OLC

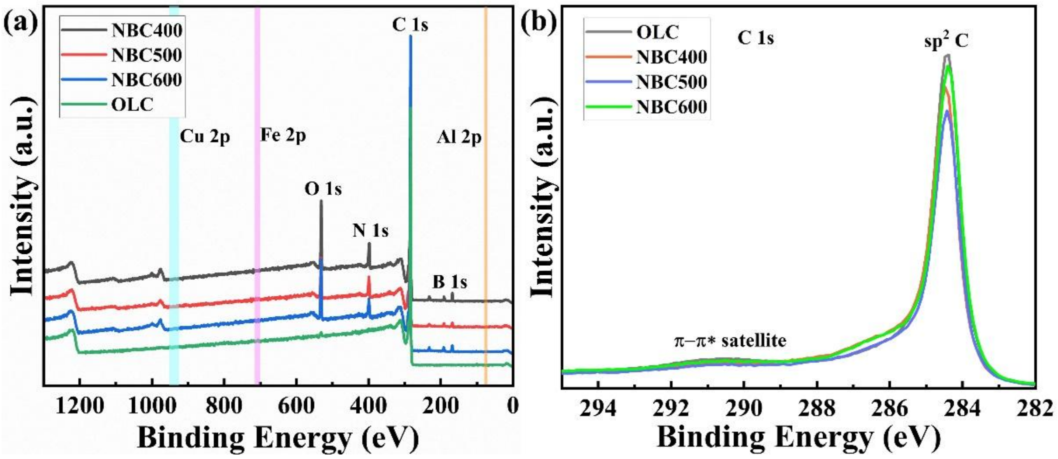

Figure S3. (a) XPS survey of samples; **(b)** C 1s XPS spectra of samples.

The N_2 and CO_2 physical adsorption of samples

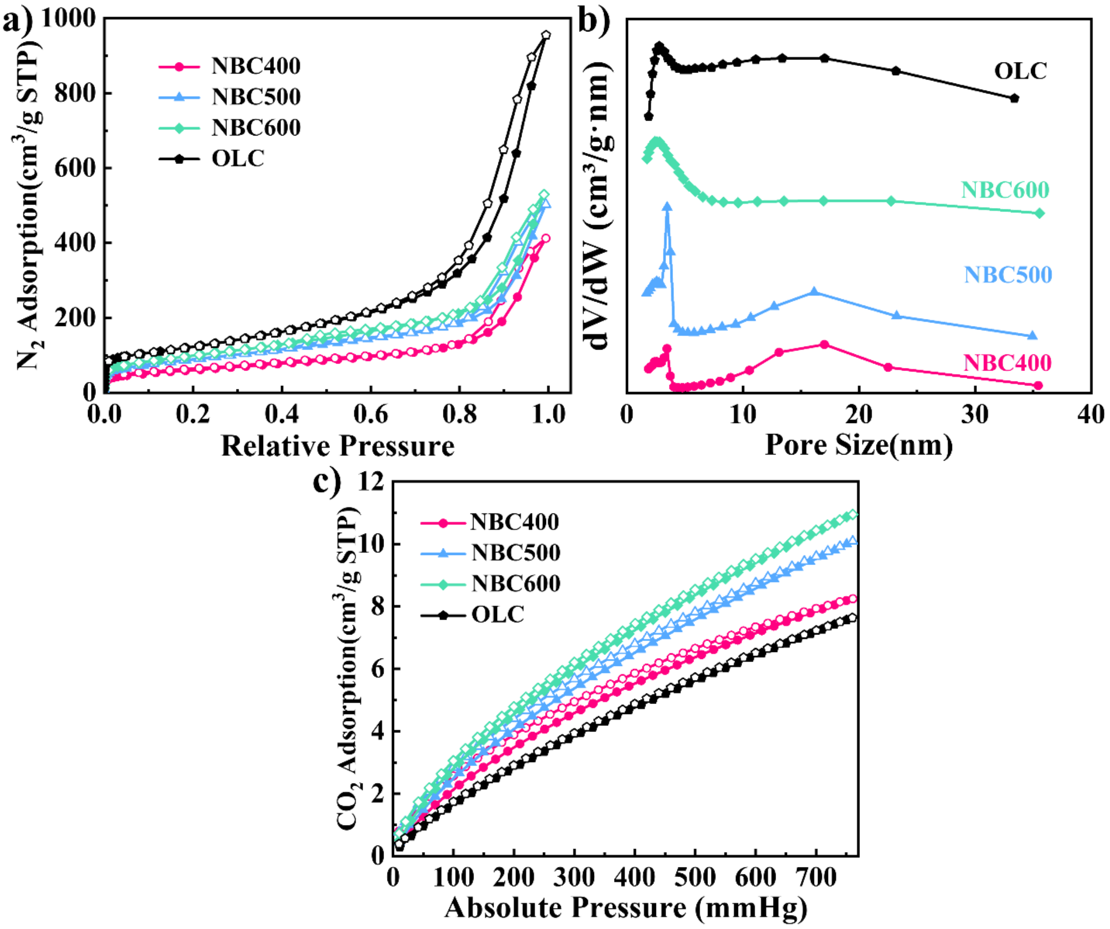

Figure S4. a) N_2 adsorption-desorption isotherms of OLC and NBC*x* samples; **b)** pore distribution of OLC and NBC*x* samples; **c)** CO_2 adsorption-desorption isotherms of OLC and NBC*x* samples.

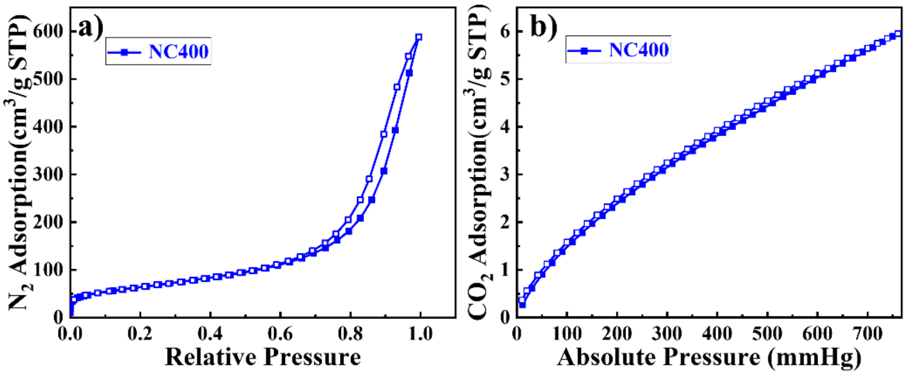

Figure S5. The N_2 **a)** and CO_2 **b)** adsorption-desorption isotherms of NC400

Physical N_2 adsorption was performed to investigate the specific surface area and pore structure of the NBC*x* samples. The N_2 adsorption-desorption isotherms and pore size distributions are shown in **Figure S4 a)** and **b)**. The N_2 adsorption-desorption isotherms and pore size distributions exhibit a Type IV adsorption

behaviour. The hysteresis loops are relatively small, and show no obvious turning point and can be classified between H3 and H4.[8] This indicates the presence of mesopores with irregular pore shapes. The surface area and pore volume all decrease after the N, B co-doping compared to OLC as shown in **Table S1**, while the surface area and pore volume of NBC*x* samples increase with the preparation temperature.

Physical CO_2 adsorption was then carried out to investigate CO_2 adsorption capacity of NBC*x* and the CO_2 adsorption-desorption isotherms are shown in **Figure S4 c)**. The adsorption-desorption isotherms exhibit a Type I adsorption behaviour, which is typical for materials with small surfaces and the formation of a monolayer adsorption in micropores.[8] It is worth noting that the amount of CO_2 adsorbed on the NBC*x* samples (0.37~0.49mmol CO_2) is all larger than that on OLC (0.34 mmol CO_2) even though OLC has the largest surface area and pore volume. More interestingly, the CO_2 desorption curves of the NBC*x* samples are slightly shifted above the CO_2 adsorption curves, whereas, the CO_2 adsorption-desorption curves of OLC are almost completely superimposed. These results indicate that there exists rather strong interaction between CO_2 and NBC*x* samples, considering the similar pore structure of OLC and NBC*x* samples smaller than 10 nm. To highlight the importance of N, B co-doping on the CO_2 adsorption, physical adsorption of N_2 and CO_2 on nitrogen doped NC400 were also carried out and the adsorption-desorption isotherms are shown in **Figure S5 a)** and **b)**.

Table S1. Pore structure and elemental composition of samples

Sample	Surface area [m²/g]	Pore Volume [cm³/g]	C [at. %]	O [at. %]	N [at. %]	B [at. %]
OLC	435.7	1.48	99.0	1.0	-	-
NBC400	217.7	0.64	83.4	7.7	6.3	2.6
NBC500	322.8	0.78	85.7	6.2	5.4	3.0
NBC600	357.3	0.82	85.4	7.1	4.3	3.3

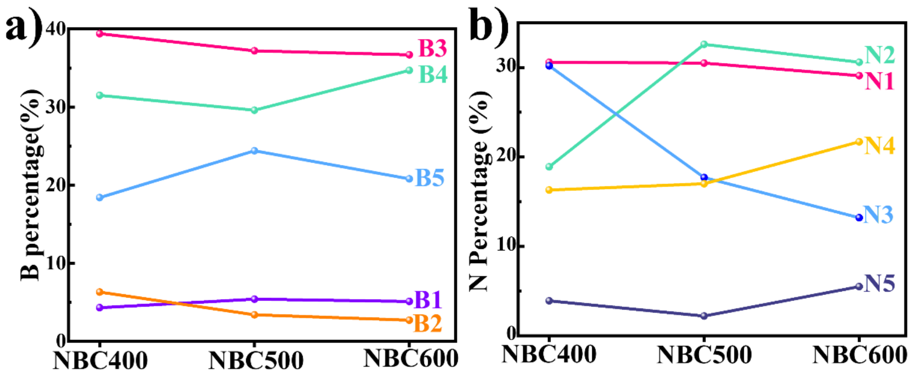

Figure S6. a) The change in B percentage of different species for NBC*x* samples; **b)** the change in N percentage of different species for NBC*x* samples.

Considering the evolution of N and B species in combination with reported carbonization process of BMIM·DCN,[9] the formation process of N, B co-doped structure could be illustrated. BMIM·DCN would be first decomposed with the elimination of alkyl chains and then condensed via the trimerization of nitrile groups, thus forming carbon debris containing triazine rings. The remaining imidazolium cationic ring would be then incorporated into carbonous debris through the nucleophilic attack of the DCN^- anion. Meanwhile, borane would decompose with the elimination of hydrogen and B would be incorporated into the carbon skeleton through reacting with carbon edge or N doping sites. As the preparation temperature increases, the elimination of hydrogen, nitrogen and alkyl fragments becomes more pronounced, and the N content decrease at the same time. While the B content remained steady in the carbon structure at nanocarbon surface due to the low volatility of its derivatives. Meanwhile, the restructuring of the carbonaceous debris occurs to fit the requirements of the locally graphitized structure as the temperature increases. The difference in the percentage of N5 species between NBC400 and NBC600 is not significant. The absolute contents of N5, calculated from the total nitrogen content and the relative N5 percentage, are also quite similar for both samples. This is likely due to the thermal stability of pyridinic N-oxide species under the synthesis conditions.

UPS spectra of samples

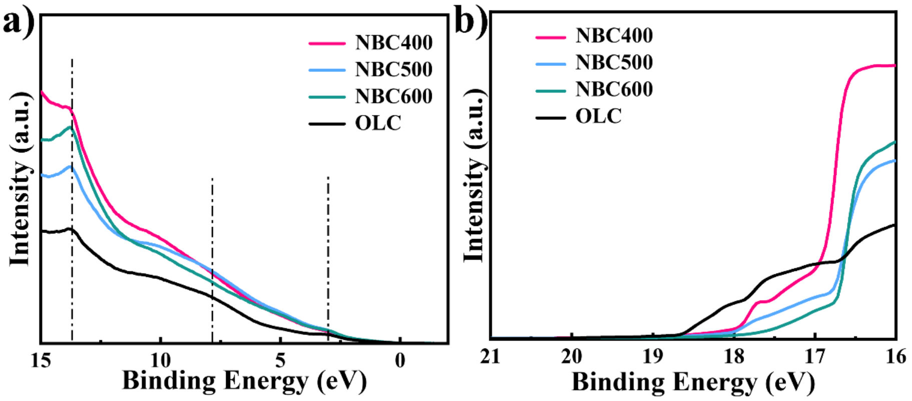

Figure S7. a) the UPS spectra of OLC and NBC*x* samples after Fermi level correction; **b)** the secondary electron tail threshold regions of UPS spectra of OLC and NBC*x* samples.

UPS spectra of samples were also collected on Thermo Scientific NEXSA Surface Analysis system with a He discharge lamp as excitation source (He I: hν = 21.22 eV).

As shown in **Figure S7 a)**, there exist three peaks at 3 eV, 7.5 eV and 13.5 eV for all samples, resulting from the 2p π, 2p σ and 2s σ electron, respectively[10]. The 2p π peak of OLC and NBC*x* samples indicates their graphitic structure. While the less prominent 2p π peaks of NBC*x* samples might be ascribed to the B doping with its all three valence electrons participating in the σ bonding with neighbouring carbon or nitrogen[11]. A steeper increase in density of states could be observed for NBC*x* samples than OLC in the region of 3~7eV, which could be attributed to the doping of electron-rich N[12]. The 2s σ peaks of NBC*x* samples increase obviously compared with that of OLC. All these results suggest the successful N, B co-doping. The secondary electron tail threshold (E_{cutoff}) regions of pristine of OLC and NBC*x* samples are shown in **Figure S7 b)**. The E_{cutoff} of the NBC*x* samples is shifted to lower binding energy in comparison to that of OLC, indicating that NBC*x* samples possess larger work function than OLC. Among NBC*x* samples, NBC400 exhibits the lowest work function suggesting the minimum energy is needed for inner electrons to escape from their nuclei on NBC400 surface.

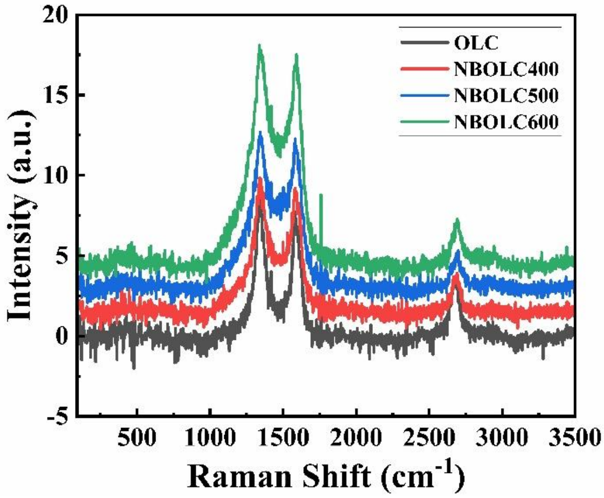

Figure S8. Raman spectra of OLC and NBC*x* samples over the full spectral range

In-situ Pyrrole and Pyridine Adsorption on N, B co-doped nanocarbon

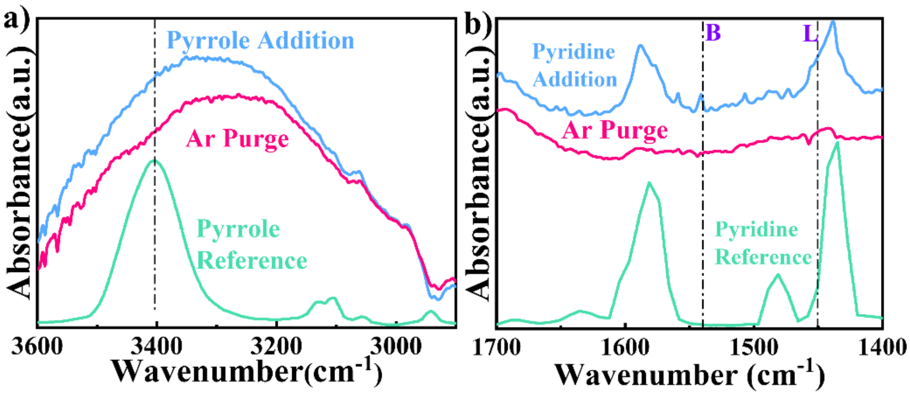

Figure S9. a) The IR spectra of pyrrole adsorption on NBC400; **b)** the IR spectra of pyridine adsorption on NBC400.

The in-situ FTIR of pyrrole and pyridine adsorption on NBC were performed based on a procedure similar to that described in the Characterization section in Experimental Section. Heteroatom doped nanocarbon materials can exhibit basicity or acidity due to the electronegativity difference between heteroatoms and carbon.[13] Pyrrole and pyridine are selected here as probe molecules to investigate the basicity and acidity of NBC*x* samples by in-situ IR technique. Pyrrole was introduced into the DRIFT cell by Ar bubbling until the IR spectra reached steady state, collected as **Pyrrole Addition**, and then purged with Ar to remove the gaseous or physically adsorbed pyrrole until the IR spectra reached steady state, collected as **Ar Purge** in **Figure S9 a)**. There are IR peaks at about 3300 cm^{-1} attributed to N-H stretching vibration for pyrrole adsorbed on NBC400, different from the gaseous or physically adsorbed pyrrole (3400 cm^{-1} in **Pyrrole Reference**). The wavenumber red shift of v_{N-H} suggests the basicity of NBC*x*, possibly due to the nitrogen doping. The prominent IR peak even after Ar purging indicate a rather strong interaction between pyrrole and NBC*x*.[14] Subsequently, the in-situ IR of pyridine adsorption was performed to investigate the acidity of NBC*x* where there would be IR peaks at about 1450cm^{-1} and 1540cm^{-1} for pyridine adsorbed on Lewis and Brönsted acidic sites.[15] Similarly, pyridine was first introduced into the DRIFT cell by Ar bubbling until the IR spectra reached steady state, collected as **Pyridine Addition**, and then purged by Ar to remove the gaseous or physical adsorbed pyridine until the IR spectra reaching steady, collected as **Ar Purge** in **Figure S9 b)**. It is obvious that there are no IR peaks at 1450cm^{-1} or 1540cm^{-1} for the spectrum of pyridine adsorbed on NBC400, and the peaks at about 1580cm^{-1} and 1440cm^{-1} from the gaseous or physically adsorbed pyridine almost disappeared after Ar purging, suggesting the weak interactions between pyridine and NBC*x* and the absence of acidity. This could be attributed to the saturated covalent bonding of the doped B atoms and the neutralization by the doped N atoms.

Construction and optimization of NBC models

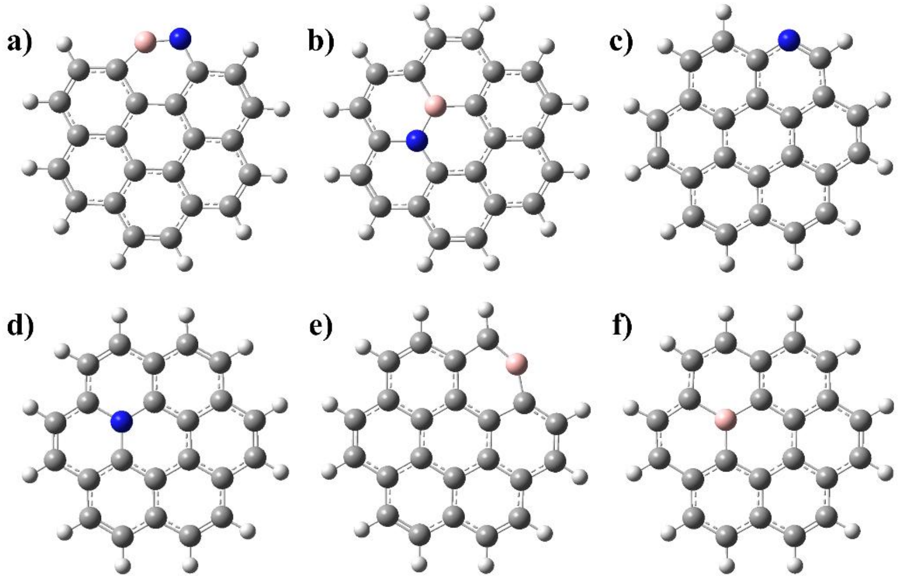

Figure S10. a) Nitrogen-boron co-doped model with N-B site at the edge (**NBC-E**); **b)** nitrogen-boron co-doped model with N-B site at the interior (**NBC-I**); **c)** nitrogen doped model with N site at the edge (**NC-E**); **d)** nitrogen doped model with N site at the interior (**NC-I**); **e)** boron doped model with B site at the edge (**BC-E**); **f)** boron doped model with B site at the interior (**BC-I**).

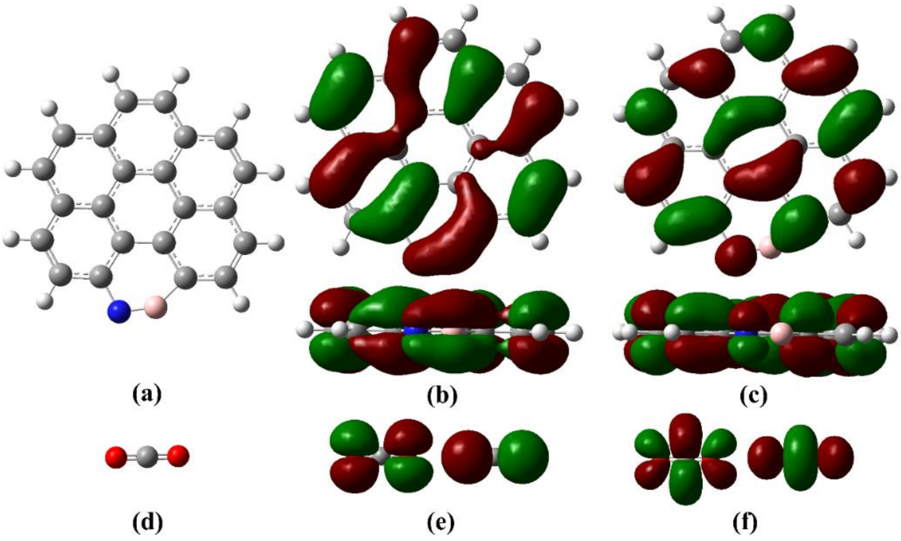

Figure S11. a) Optimized structure of NBC model; **b)** highest occupied molecular orbital (HOMO) of NBC with energy of -0.32183 Ha(top view(top), side view(bottom)); **c)** lowest unoccupied molecular orbital (LUMO) of NBC with energy of -0.22920 Ha(top view(top), side view(bottom)); **d)** optimized structure of CO_2; **e)** HOMO of CO_2 with energy of -0.41858 Ha(top view(left), side view(right)); **f)** LUMO of CO_2 with energy of -0.06683 Ha(top view(left), side view(right)).

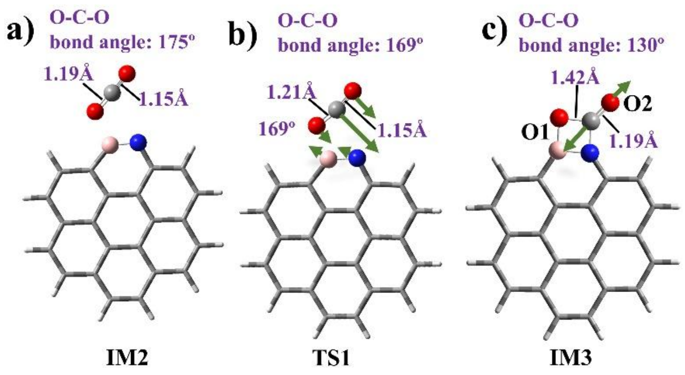

Figure S12. The structure and parameters of **a) IM2** (CO_2 physisorption on NBC), **b) TS1** (transition state from CO_2 physisorption to chemisorption with the olive arrow indicating the vibration vector corresponding to the sole imaginary frequency) and **c) IM3** (CO_2 chemisorption on NBC model with the olive arrow indicating the C=O stretching vibration vector of the highest intensity).

Liquid Product Analysis

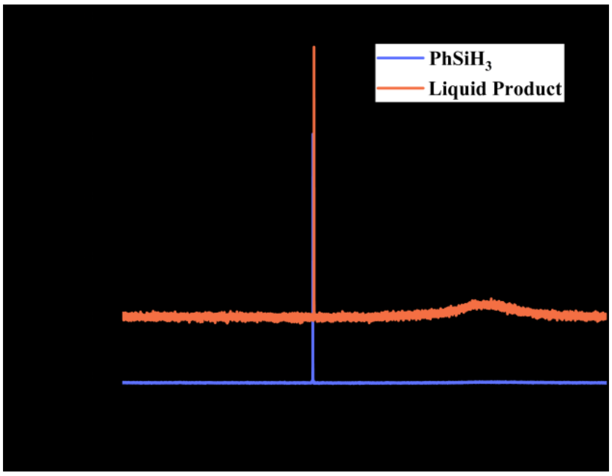

Figure S14. ^{29}Si NMR signal of fresh $PhSiH_3$ and reaction liquid product. Reaction condition: 10 mg NBC400, 1 mL dodecane as solvent, 2mmol $PhSiH_3$, 4 MPa CO_2, 12h, stirring speed of 800 rpm.

For fresh $PhSiH_3$, there is a single peak at -60 ppm in the ^{29}Si NMR. While for that of the liquid product, in addition to the signal from unreacted $PhSiH_3$, a broad peak appears in the range of -100 to -120 ppm, which can be attributed to oxidation products of $PhSiH_3$. During the reaction, the C=O bond of CO_2 is cleaved with the assistance of the catalyst and $PhSiH_3$, whereby the oxygen atom is captured by silicon to form siloxane species. Given the high reaction temperature, it is reasonable to deduce that these siloxanes would undergo further condensation to form silicones. Previous studies have shown that the ^{29}Si NMR chemical shift of condensed silicones appears at lower fields (more negative values) compared to their parent siloxanes. Considering the ^{29}Si NMR chemical shift of triethoxyphenylsilane (-60.5ppm) and $PhSiH_3$(-60ppm), the broad peak observed between -100 and -120 ppm can be reasonably assigned to condensed silicone species.

Catalysts Stability Test

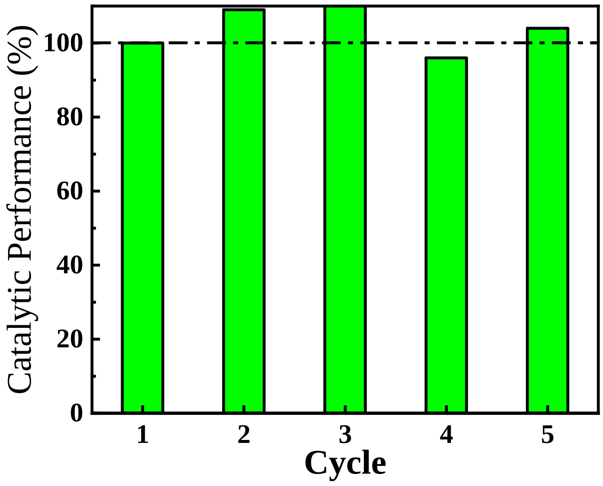

Figure S13. Recycling performance of NBC400 (the catalytic performance in the recycling experiments is normalized by that of fresh catalyst). Reaction condition: 20 mg catalyst, 2 mL toluene as solvent, 1 mmol $PhSiH_3$, 4 MPa CO_2, 180 ^oC, 8 h. After each reaction, the catalyst was separated from the reaction medium through centrifugation and washed with toluene for the next cycle.

IR signals of $PhSiH_3$ on OLC

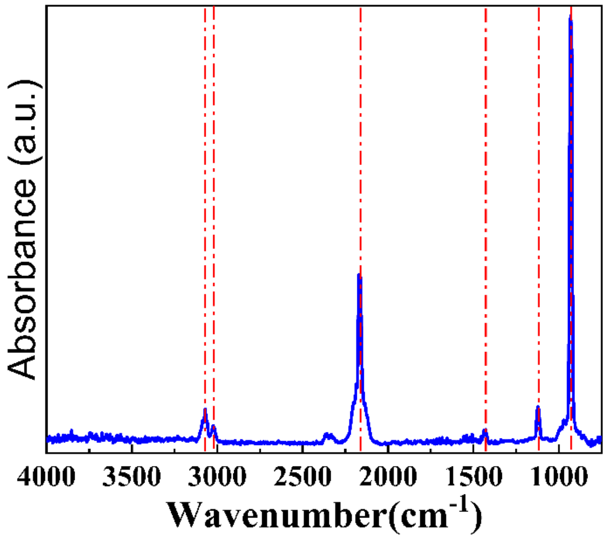

Figure S14. The IR signals of $PhSiH_3$

First the intrinsic IR spectrum of $PhSiH_3$ without the disturbance from the functional surface was collected as benchmark for further analysis in **Figure S14**. Here, $PhSiH_3$ was blew into the DRIFT cell containing OLC by the flow of Ar and its corresponding IR spectrum was obtained. The characteristic IR peaks in this spectrum mainly at 930 cm^{-1}, 1123 cm^{-1}, 2171 cm^{-1}, 3100~2950 cm^{-1} correspond to Si-H deformation vibration ($\delta_{Si\text{-}H}$), C-H in-plane bending vibrations ($\beta_{C\text{-}H}$), Si-H stretching vibrations ($v_{Si\text{-}H}$), C-H stretching vibrations ($v_{C\text{-}H}$), respectively.

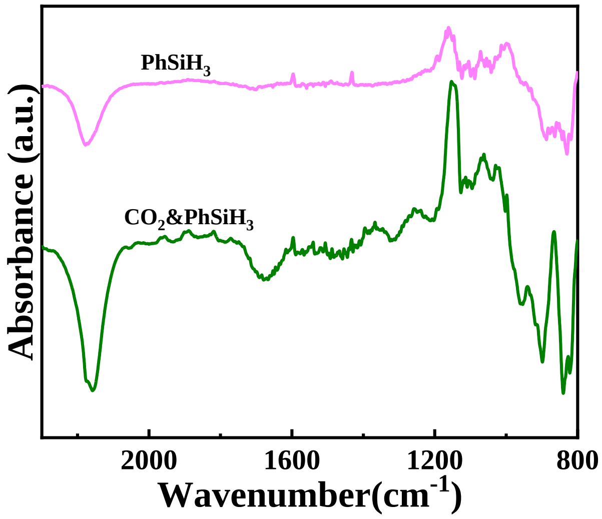

Figure S15. The differential spectrum of $PhSiH_3$ adsorption on NBC400 between 40^oC and 180^oC in comparation with that of $PhSiH_3$ adsorption on CO_2 adsorbed NBC400.

The possibility of desorption of adsorbed $PhSiH_3$ from fresh NBC400 was also considered whose downward peak is rather weaker than that from CO_2 adsorbed NBC400 in **Figure S15**, confirming the reaction of chemisorbed CO_2 with activated $PhSiH_3$.

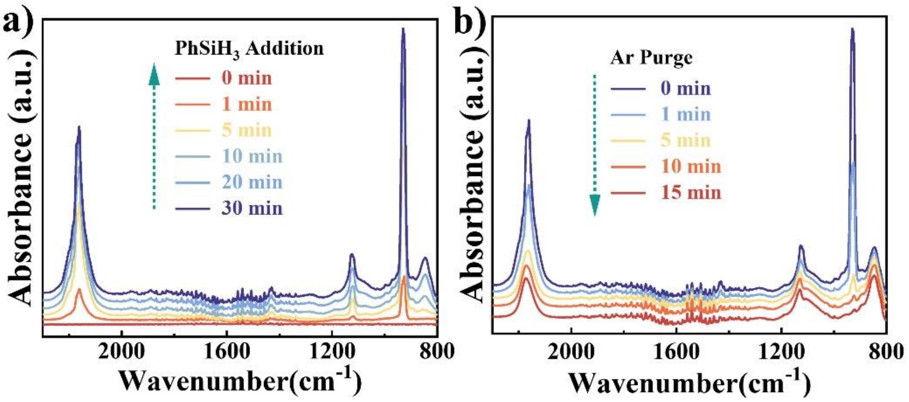

Figure S15. a) The in-situ FTIR spectra of $PhSiH_3$ addition into fresh NBC400 at wavenumber of 2240~800 cm^{-1}; **b)** in-situ FTIR spectra of Ar purge after $PhSiH_3$ addition into fresh NBC400 at wavenumber of 2240~800 cm^{-1}.

To gain more insights into the reaction mechanism, in-situ FTIR of $PhSiH_3$ addition followed by CO_2 addition was further performed. During the $PhSiH_3$ addition into fresh NBC400, the IR peaks at 930 cm^{-1} and 2171 cm^{-1} from $PhSiH_3$ itself also increase with the bubbling time as shown in **Figure S15 a)**. After the in-situ FTIR spectra of $PhSiH_3$ addition reached steady, Ar purging was started to blow away the physically adsorbed $PhSiH_3$ during which the IR peak at 2171 cm^{-1} decreased and IR peak at 930cm^{-1} disappeared finally like that of the $PhSiH_3$ addition on CO_2 adsorbed NBC400.

References

[1] aY. Lin, Z. Liu, L. Yu, G. R. Zhang, H. Tan, K. H. Wu, F. Song, A. K. Mechler, P. P. M. Schleker, Q. Lu, B. Zhang, S. Heumann, *Angew. Chem. Int. Ed.* **2021**, *60*, 3299-3306; bY. Lin, X. Sun, D. S. Su, G. Centi, S. Perathoner, *Chem Soc Rev* **2018**, *47*, 8438-8473; cM. Zeiger, N. Jäckel, V. N. Mochalin, V. Presser, *J Mater Chem A* **2016**, *4*, 3172-3196.

[2] M. Cui, Q. Qian, J. Zhang, Y. Wang, B. B. Asare Bediako, H. Liu, B. Han, *Chem* **2021**, *7*, 726-737.

[3] aM. J. Frisch, G. W. Trucks, H. B. Schlegel, G. E. Scuseria, M. A. Robb, J. R. Cheeseman, G. Scalmani, V. Barone, G. A. Petersson, H. Nakatsuji, X. Li, M. Caricato, A. V. Marenich, J. Bloino, B. G. Janesko, R. Gomperts, B. Mennucci, H. P. Hratchian, J. V. Ortiz, A. F. Izmaylov, J. L. Sonnenberg, D. Williams-Young, F. Ding, F. Lipparini, F. Egidi, J. Goings, B. Peng, A. Petrone, T. Henderson, D. Ranasinghe, V. G. Zakrzewski, J. Gao, N. Rega, G. Zheng, W. Liang, M. Hada, M. Ehara, K. Toyota, R. Fukuda, J. Hasegawa, M. Ishida, T. Nakajima, Y. Honda, O. Kitao, H. Nakai, T. Vreven, K. Throssell, J. A. Montgomery, J. E. P. Jr., F. Ogliaro, M. J. Bearpark, J. J. Heyd, E. N. Brothers, K. N. Kudin, V. N. Staroverov, T. A. Keith, R. Kobayashi, J. Normand, K. Raghavachari, A. P. Rendell, J. C. Burant, S. S. Iyengar, J. Tomasi, M. Cossi, J. M. Millam, M. Klene, C. Adamo, R. Cammi, J. W. Ochterski, R. L. Martin, K. Morokuma, O. Farkas, J. B. Foresman, D. J. Fox, Revision A.03 ed., Gaussian, Inc., Wallingford CT, **2016**; bS. Grimme, J. Antony, S. Ehrlich, H. Krieg, *Journal of Chemical Physics* **2010**, *132*; cS. Grimme, S. Ehrlich, L. Goerigk, *Journal of computational chemistry* **2011**, *32*, 1456-1465.

[4] aH. Choi, Y. C. Park, Y. H. Kim, Y. S. Lee, *J Am Chem Soc* **2011**, *133*, 2084-2087; bS. Vuckovic, K. Burke, *The journal of physical chemistry letters* **2020**, *11*, 9957-9964; cM. Wang, X. He, M. Taylor, W. Lorpaiboon, H. Mun, J. Ho, *J Chem Theory Comput* **2023**, *19*, 5036-5046.

[5] aF. Neese, *WIREs Computational Molecular Science* **2017**, *8*; bF. Weigend, R. Ahlrichs, *Phys Chem Chem Phys*

2005, *7*, 3297-3305.

[6] T. Lu, F. Chen, *Journal of computational chemistry* **2012**, *33*, 580-592.

[7] P. Zhang, B. Yang, H. Ma, Z. Wu, *Fuel* **2021**, *293*.

[8] K. S. W. Sing, D. H. Everett, R. A. W. Haul, L. Moscou, R. A. Pierotti, J. Rouquerol, T. Siemieniewska, *Pure Appl Chem* **1985**, *57*, 603-619.

[9] aT. Du, P. Zhang, G. Wang, Z. Jiao, J. Zhou, Y. Ding, *Nanoscale* **2025**, *17*, 1392-1399; bJ. P. Paraknowitsch, J. Zhang, D. Su, A. Thomas, M. Antonietti, *Adv Mater* **2010**, *22*, 87-92.

[10] Y. Lin, Z. Feng, L. Yu, Q. Gu, S. Wu, D. S. Su, *Chem Commun* **2017**, *53*, 4834-4837.

[11] Y. Lin, Y. Zhu, B. Zhang, Y. A. Kim, M. Endo, D. S. Su, *J Mater Chem A* **2015**, *3*, 21805-21814.

[12] S. H. Lim, H. I. Elim, X. Y. Gao, A. T. S. Wee, W. Ji, J. Y. Lee, J. Lin, *Phys Rev B* **2006**, *73*.

[13] Y. Ding, X. Huang, X. Yi, Y. Qiao, X. Sun, A. Zheng, D. S. Su, *Angew. Chem. Int. Ed.* **2018**, *57*, 13800-13804.

[14] J. C. Lavalley, *Catal Today* **1996**, *27*, 377-401.

[15] M. Tamura, K.-i. Shimizu, A. Satsuma, *Applied Catalysis A: General* **2012**, *433-434*, 135-145.
